# Case report: eosinophilic myocarditis in hypereosinophilic syndrome: a journey to heart transplantation

**DOI:** 10.3389/fimmu.2024.1418665

**Published:** 2024-06-07

**Authors:** Shriya Sharma, Smruti Desai, Juan Leoni, Smit Paghdar, Jose Ruiz, Rohan Goswami

**Affiliations:** Department of Cardiology, Division of Advanced Heart Failure and Transplant Cardiology, Mayo Clinic, Jacksonville, FL, United States

**Keywords:** hypereosinophilia, myocarditis, heart failure, restrictive cardiomyopathy, heart transplant

## Abstract

**Introduction:**

Hypereosinophilic Syndrome (HES) is a rare disorder characterized by persistent elevation of eosinophils, leading to multi-organ infiltration and damage. Eosinophilic Myocarditis (EM) is one of its severe complications contributing significantly to morbidity and mortality. Herein, we describe the diagnostic and therapeutic challenges of EM, emphasizing the significance of early recognition and multidisciplinary management.

**Case presentation:**

A 51-year-old female with a history of EM, heart failure, and peripheral eosinophilia presented with NYHA class 3b symptoms. Laboratory findings revealed elevated peripheral eosinophil count, NT-Pro BNP, and characteristic electrocardiogram abnormalities. Imaging studies confirmed biventricular thrombi and myocardial abnormalities consistent with EM. Treatment involved Solu-Medrol for HES and heparin for ventricular thrombi, leading to initial clinical improvement. However, refractory heart failure necessitated urgent heart transplantation.

**Discussion:**

EM, an under-recognized complication of HES, poses diagnostic and management challenges. Management includes standard heart failure treatments, steroids, and emerging therapies like Mepolizumab. Early diagnosis and aggressive management are pivotal for improving outcomes in this rare and potentially fatal condition.

**Conclusion:**

Advancements in the detection of complications, surgical management, and therapeutic options have improved outcomes in HES. Ongoing research is essential to further understand and address the diagnostic and therapeutic challenges of HES and EM.

## Introduction

Hypereosinophilic syndrome (HES) is characterized by unexplained continuous overproduction of eosinophils, resulting in multi-organ eosinophilic infiltration, eventually leading to organ damage and dysfunction. The first diagnostic criteria for HES are defined as (1) absolute eosinophil count (AEC) ≥ 1500/μL for longer than six months (or death before six months associated with signs and symptoms of hypereosinophilic disease), (2) absence of parasitic, allergic, or other known causes of eosinophilia, and (3) signs of organ involvement, such as heart failure, gastrointestinal dysfunction, central nervous system abnormalities, fever, or weight loss (1). Skin, lung, and gastrointestinal tract are commonly involved organs in patients with HES. Less commonly, it also affects the cardiovascular system and brain, which could be fatal. Clinical presentation of HES varies from incidental findings on laboratory investigations to life-threatening conditions, as outlined above. In many patients, the onset of symptoms is insidious due to the rapid symptoms of cardiovascular and neurologic complications.

Cardiovascular complications are a significant cause of morbidity and mortality among patients with HES (2). Recent studies suggest that 40% to 50% of patients with HES have signs and symptoms of cardiac involvement (3). Eosinophil-mediated cardiac injuries are divided into three pathological phases: acute early necrosis due to eosinophilic infiltration followed by myocarditis, thrombi formation due to damaged endocardium, and fibrosis, which leads to restrictive cardiomyopathy (RCM) from Eosinophilic myocarditis (EM) (4).

EM is a rare, often under-recognized, and fatal complication of HES if left untreated (5). Early recognition and appropriate treatment are crucial for improving patient outcomes. Recent literature shows a gradually increasing diagnostic rate with the advancements in cardiac imaging and endomyocardial biopsy (6). Due to the rare prevalence, we felt it imperative to describe the diagnostic pathway and treatment algorithms of EM in a patient who eventually underwent heart transplantation due to her disease process.

## Case presentation

A 51-year-old Caucasian female with a history of heart failure with mid-range ejection fraction (41%) due to EM, cerebrovascular accident, residual right-sided lower extremity weakness, asthma, recurrent bronchitis, ventricular tachycardia, right ventricular thrombus, and peripheral eosinophilia with concern for HES was referred for advanced therapies. Her complaints centered around fatigue and dyspnea with minimal exertion, confusion, poor appetite, and orthopnea and paroxysmal nocturnal dyspnea – all consistent with NYHA class 3b functional status. At presentation, her temperature was 35.8°C, with a blood pressure of 94/67 (76) mmHg, a heart rate of 61 bpm, with SpO2 of 94% on room air. Physical examination was notable for a distended abdomen with hepatomegaly (liver edge palpable 2 cm below costal margin) and cool extremities to touch, consistent with Stevenson profile C. We attempted to optimize the patient’s guideline-directed medical therapy, salt, and fluid balance, as an outpatient. However, she was intolerant. Her refractoriness resulted in progressive hypotension and confusion. One month after her initial visit, she was admitted to managing and treating acute decompensated heart failure.

**Figure 1:**
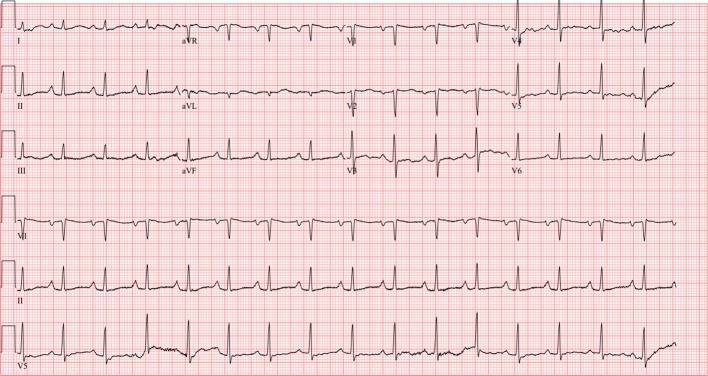
EKG showing sinus rhythm with 1st-degree A-V block, right atrial enlargement, nonspecific T wave abnormality, and prolonged QT and PR intervals.

During her admission, laboratory workup demonstrated a peripheral eosinophil count of 1.77(NR 0.01 - 0.08 cells/uL), total eosinophils of 15.4% (NR 1.0-3.0%), absolute eosinophil count of 4.18 (0.03 to 0.48 cells/ul) and NT-Pro B-type natriuretic peptide (NT-Pro BNP) of 1723 pg/mL (NR <144 pg/mL). There was no significant troponin elevation (<.01). Electrocardiogram (EKG) showed sinus rhythm with 1st-degree A-V block, right atrial enlargement, nonspecific T wave abnormality, and prolonged QT and PR intervals, unchanged from outside hospital records (**Figure 1**).

**Figure 2:**
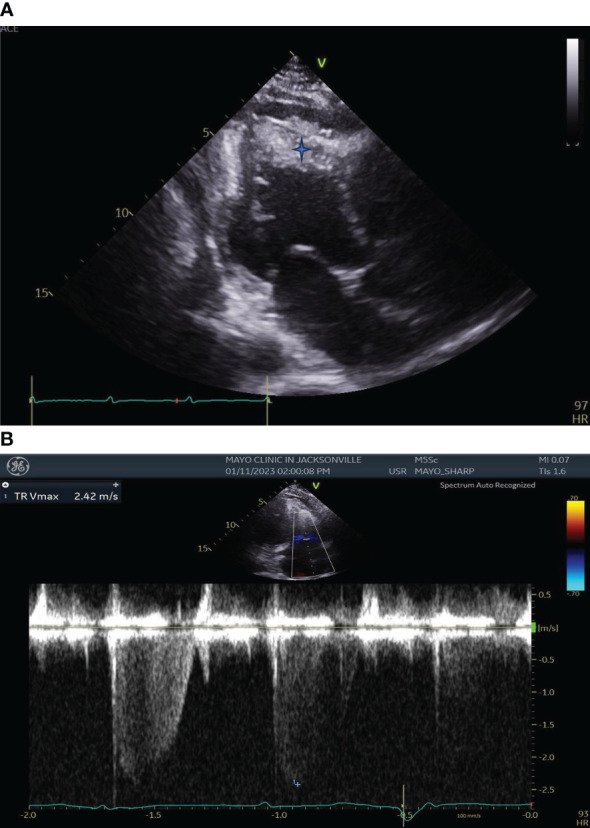
**(A)** Transthoracic Echocardiography showing left ventricular wall motion abnormalities, enlarged right ventricular chamber size with biventricular apical thrombi (*Blue star)*. **(B)** Transthoracic Echocardiography showing severe Tricuspid Regurgitation.

Transthoracic echocardiography (TTE) demonstrated left ventricular wall motion abnormalities with an ejection fraction of 41%, moderately enlarged right ventricular chamber size with reduced systolic function, severe tricuspid valve regurgitation, and biventricular apical thrombi (**Figure 2**).

Given her intolerance to medical therapy, a peripherally inserted central catheter (PICC) was utilized to obtain a central mixed venous saturation (ScVO2) of 42%, yielding a Fick cardiac output of 1.80 L/min and an index of 1.01 L/min/m2. Given the evidence of NYHA 3b, AHA D, Stevenson profile C cardiogenic shock, she was started on dobutamine 2.5 mcg/kg/min and Bumex IV 2.5 mg BID.

**Figure 3:**
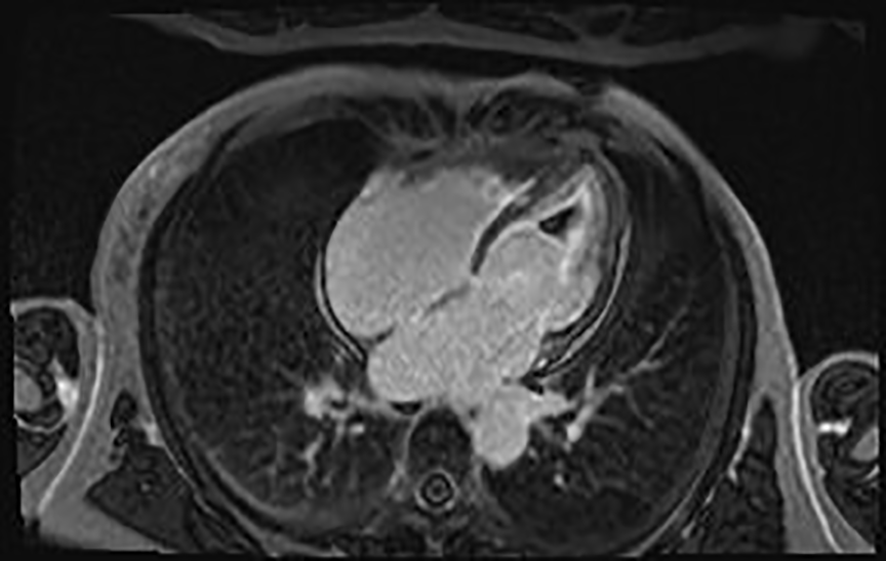
Cardiac MRI with contrast showing diffuse mid to apical left ventricular subendocardial abnormal late gadolinium enhancement.

Cardiac MRI (CMR) with and without contrast showed diffuse mid to apical left ventricular subendocardial abnormal late gadolinium enhancement, patchy mid-wall enhancement, and areas of focal enhancement of the mid-anterior and inferior/inferolateral walls consistent with fibrosis (**Figure 3**).

A bone marrow biopsy was performed, which showed hypocellular marrow (~25%) with trilineage hematopoiesis and hypereosinophilia with 50% increase of eosinophils with no evidence of dysplasia. Fluorescence *in situ* hybridization (FISH) analysis was negative for BCR-ABL, F GFR 1. FISH analysis also produced negative results for abnormalities related to PDGFRA, PDGFRB, FGFR1, and t (9:22). Peripheral blood flow cytometry also returned negative findings, ruling out leukemia/lymphoma. Autoimmune serology was negative. Viral research, stool test for strongyloides and parasites were negative too.

Immunohistochemical stains for CD34 and CD117 showed no increase in blasts. MPO highlighted granulopoiesis. CD3 demonstrated a normal complement of T-cells, while PAX-5 showed a normal complement of B-cells. The reticulin stain was focally borderline. Flow cytometry detected no increase in blasts, no monotypic B-cell populations, and no phenotypically aberrant T-cell populations.

The evaluation of blasts revealed no increase in their numbers. Analysis of B-cells showed no presence of monotypic populations and exhibited a normal expression pattern of CD19, CD10, surface kappa, and lambda. T-cells/NK-cells did not display any aberrant phenotype based on CD3 and CD16 markers. The bone marrow biopsy indicated normocellular morphology, with a 50% increase in eosinophils. Importantly, no signs of lymphoma or mass disease were observed. Flow cytometry results were consistently negative, indicating no increase in plasma cells. Further testing through FISH analysis produced negative results for abnormalities related to PDGFRA, PDGFRB, FGFR1, and t(9:22). Peripheral blood flow cytometry also returned negative findings, ruling out leukemia/lymphoma.

The patient was started on Solu-Medrol 1000 mg IV daily x 3 days to treat HES and heparin infusion for ventricular thrombi. Subsequently, her condition improved, and the patient was weaned from dobutamine based on daily ScVO2 and Fick calculations and successfully transitioned to oral therapies.

EMB was initially not performed because of elevated risk of complications. Given the long- term complications and risk of death with EM the patient was started on steroids as an outpatient after clinical suspicion was noted, and biopsy was scheduled to be done thereafter. She had a cardiac MRI which demonstrated findings consistent with eosinophilic myocarditis and did have peripheral eosinophilia.

**Figure 4:**
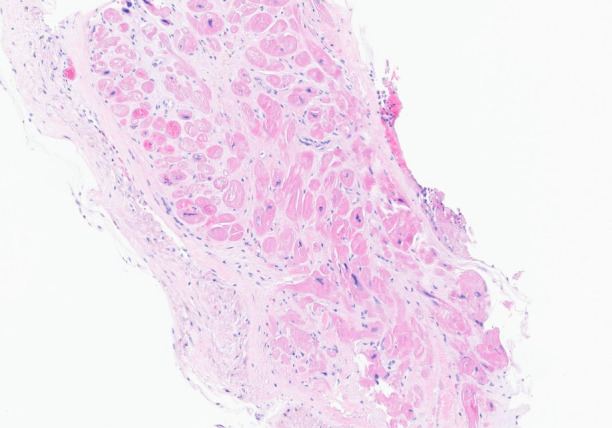
Endomyocardial biopsy showing no lymphocytic or eosinophilic infiltration or granuloma formation.

An endomyocardial biopsy was performed after steroids were given and was negative for lymphocytic or eosinophilic infiltration or granuloma formation (**Figure 4**). Congo red stain was negative for amyloid. The iron stain was negative for abnormal iron accumulation, and the trichrome stain was negative for significant interstitial fibrosis.

Clinical history, in combination with imaging data from echocardiography and CMR, was highly suggestive of eosinophilic myocarditis. Her autoimmune serology, including the ANA test, has previously showed negative results. Testing for hepatitis B, hepatitis C, and HIV has also been negative in the past. Additionally, stool tests for strongyloides and ova and parasites were negative. Therefore, our case was diagnosed as Idiopathic Hypereosinophilia, indicating elevated eosinophil levels without a known cause associated with end-organ damage.

The best clinical treatment of HES depends on disease etiology and subtypes. However, even in the absence of a known cause, HES must be promptly treated in order to reduce potential morbidity that can result from organ damage.

This patient was treated with pulse steroids and heparin infusion for ventricular thrombi. Clinical condition improved and discharged with therapy with steroid, bumetanide and apixaban.

The patient was discharged on oral prednisone taper, Bumex orally 2mg daily, and Eliquis 5mg twice daily. She started on Mepolizumab (*Nucala, GlaxoSmithKline LLC*) 300 mg subcutaneously every four weeks. Since discharge, unfortunately, the patient had refractory heart failure off Dobutamine and required readmission. She was urgently listed for heart transplantation. The donor was identified as an increased risk for transmitting blood-borne illnesses. Serologies indicated that the donor was positive for CMV and EBV, matching the recipient’s positive status for both. The ischemic time for the transplant was 3 hours and 34 minutes. Intraoperatively, the patient received 1 unit of PRBC, 1 unit of FFP, 1 unit via Cell Saver, IV Solu-Medrol and IV CellCept.

### Post- transplant care

The patient was extubated by postoperative day 1. The postoperative course was uncomplicated. Upon discharge, the patient’s immunosuppressive regimen included: Prednisone taper at 20 mg daily, oral CellCept (Mycophenolate Mofetil) at a dose of 1000 mg twice a day. Tacrolimus (Prograf) was initiated on day 2, with a target level of 9-12. At discharge, the patient’s Tacrolimus level was 6.9, and she was discharged on Prograf 5 mg twice a day.

On a 12-week follow-up she remains stable with no evidence of rejection on EMB and had no recent hospitalizations with an LVEF of 60%. Hematology felt that steroids and immunotherapy would control bone marrow eosinophilia and continue to follow her.

## Discussion

EM is a rare form of myocarditis usually associated with fever, rash, and peripheral eosinophilia (7, 8). Published literature describes only 5% of patients presenting with cardiac complaints at initial diagnosis (9). A large amount of data has been published regarding the high-risk features of EM and prognosis. However, no clear consensus documentation has been developed for the early detection, treatment, and long-term management in this population. Furthermore, data exists regarding the role of eosinophilic granulomatosis and polyangiitis with outcomes in patients after heart transplantation, demonstrating acceptable survival (10). Due to the complexity of medical therapy in advanced heart failure - and association with eosinophilia – outcomes data after heart transplant for patients with primary HES and/or EM is limited (11). Our case presentation and literature review highlight the benefits of early diagnosis and aggressive management in a multidisciplinary approach with hematology and transplant cardiology.

Below we discuss both diagnostic pathways and suggested criteria/timing of management in patients with EM or HES. We conclude by outlining novel therapeutic and diagnostic modalities that may help elucidate patient response to therapy or the need for earlier advanced heart failure management to improve survival.

### Patient presentation

Various presentations include rapidly progressive heart failure, necrotizing eosinophilic myocarditis (with or without the association of HES), or sudden death. Individuals with necrotizing eosinophilic myocarditis have a worse prognosis with a presentation of biventricular heart failure, similar to our patients. Individuals that develop ventricular arrhythmias may have an increased rate of premature death. The rarity of EM requires high levels of suspicion while evaluating patients with peripheral eosinophilia and concomitant heart failure. Unfortunately, this poses diagnostic challenges due to a lack of specific diagnostic criteria, expertise, and availability for endomyocardial biopsy to be performed safely in the non-advanced heart failure center.

Initiation of therapy is not warranted in asymptomatic individuals due to the drug’s side effects. Broadly, the diagnostic criteria of HES can be divided into emergent and non-emergent presentations.

### Diagnostic criteria and timing

Emergent presentation is guided by 1. absolute eosinophil count exceeding 100,000cells/microL, 2. presence of leukostasis, 3. symptoms of acute heart failure or symptomatic arrhythmia, 4. evidence of eosinophil-mediated cardiac damage, and 5. arterial thromboembolism causing central nervous system infarctions. In situations with high suspicion of EM, emergent therapy may be indicated (12).

While some individuals with HES require immediate treatment, asymptomatic patients can be safely monitored regularly. Treatment aims to reduce the absolute eosinophil count, mitigate signs/symptoms, and prevent disease progression (13).

Symptomatic but clinically stable — For the patient who is symptomatic and clinically stable, the urgency of evaluation depends upon the clinical presentation, level of eosinophilia, and concern on the part of the clinician and/or patient.

In general, the AEC alone should not determine the urgency of evaluation in a clinically stable patient. However, patients with ≥5000 eosinophils/microL or rapidly rising AEC should be evaluated promptly.

For other symptomatic but clinically stable patients, the urgency of evaluation is informed by findings that may reflect organ involvement and/or the cause of eosinophilia and by concerns on the part of the clinician and patient.

Asymptomatic or incidental eosinophilia — All patients with AEC ≥1500/microL should have a CBC repeated in one to two weeks to determine if the eosinophilia is transient, stable, or rising; the CBC should be repeated even when eosinophilia is detected incidentally in an asymptomatic patient. Persistent AEC >1500/microL or a rising AEC should be evaluated promptly for HES, even though it is uncommon for such patients to be completely asymptomatic.

For asymptomatic patients with eosinophilia <1500/microL, postponing a repeat CBC and evaluation for a month or longer may be reasonable. However, it is important first to ensure that there are no clinical findings suggestive of eosinophilic end-organ damage, no history of travel or residence in helminth-endemic areas, and no features suggestive of malignancy (eg, significant anemia or thrombocytopenia, splenomegaly, lymphadenopathy) before deferring the evaluation.

Consensus statements currently available outline various therapeutic options for supporting patients with both emergent and nonemergent presentations. However, there are no clear diagnostic pathways to define standardized treatment protocols in this population. Below, we outline a focused approach to managing HES-Cardiomyopathy (HESCM) patients (12).

### HESCM management

The criteria to treat patients with HES includes that they have >1500/mm3 or more eosinophils for six or more months with evident tissue damage. Non-hematologic secondary causes causing peripheral eosinophilia needs to be ruled out. The therapy for individuals diagnosed with myocarditis includes the standard treatment for heart failure. Progression of HES-CM occurs in three consecutive phases: 1) eosinophilic infiltration, 2) thrombosis 3) and eventual fibrosis, which leads to restrictive cardiomyopathy. Standard heart failure treatments, including angiotensin-converting enzyme inhibitors, angiotensin receptor blockers, and aldosterone receptor antagonists, improve myocardial remodeling, provide symptomatic relief, and prevent sudden cardiac death due to impaired cardiac function (14).

### Medical therapy

While many studies have been conducted using various treatment modalities, the standard treatment for HES-CM is steroids. In a survey conducted by Khalid et al., they used methylprednisolone 125 mg intravenous starting bolus, followed by 40 mg intravenously every eight hours. They could double their patient ejection fraction within the first three days (11). In 2017, Brambatti et al. found a lower incidence of in-hospital death among those treated with corticosteroids (9.9%) versus those who were not (65.7%) (15).

### Progressive symptoms

If conventional drug treatment is unsuccessful, cardiac support with temporary mechanical circulatory support devices can be pursued. Some examples of these devices that could be utilized include temporary left ventricular assist devices, extracorporeal membrane oxygenation, and intra-aortic balloon pump counterpulsation. Durable left ventricular assist devices are often not considered feasible due to the risk of progressive intracardiac thrombus formation or pre-formed clot, increasing the risk of stroke at LVAD placement. Right heart support may be warranted – but limited – in patients with bi-ventricular failure due to increased thrombi burden or arrhythmia.

### Next-generation therapy

In September 2020, the Food and Drug Administration (FDA) approved the first targeted biologic treatment, Nucala (mepolizumab), in both pediatric patients and adults above the age of 12 years old that have had HES (16). It is an antibody that targets IL-5 and has been shown to reduce disease flares in patients with IPL1/PDGFRA – negative HES.

The therapeutic guideline of HES depends on numerous factors, including the clinical presentation, laboratory findings, and the variant of gene mutation (FIP1L1/PDGFRA mutation), which is HES with myeloproliferative features. Individuals with the FIPL1/PDGFRA mutation have a worse prognosis and should be treated aggressively with Imatinib, a BCR-ABL protein-tyrosine kinase inhibitor.

Individuals without the mutation can be treated with the first-line therapy of glucocorticoids, a second-line therapy of hydroxyurea/interferon alpha, and a third-line drug as a high dose of Imatinib (17). Individuals with refractory cases, despite Imatinib therapy, may be treated with hematopoietic stem cell transplantation to reverse organ dysfunction. Various chemotherapeutic agents have been utilized for cases that show resistance to the usual treatment modalities.

Patients who do not respond to these therapies may be worked up for a heart transplant. Orthotopic heart transplantation can be considered for patients with advanced disease and debilitating symptoms. In cases where optimal management fails to alleviate heart failure or recurrent valve thrombosis, transplantation becomes necessary (18, 19).

## Conclusion and future directions

The prognosis of HES depends on the involvement of the heart and the increased likelihood of developing hematological malignancies. The outcomes of HES have significantly improved over time due to earlier detection of complications, better surgical management of cardiac and valvular disease, and the use of a broader and new spectrum of therapeutic molecules for controlling hypereosinophilia. Early diagnosis and aggressive management may improve outcomes in this rare disease. However, a heightened sense of consideration should be part of the clinician evaluating patients with abnormal eosinophil counts and concern for cardiac dysfunction. Future directions with novel therapeutics, biomarkers, and prognostic assessment provide increased hope for patients stricken with this fatal condition.

## Data availability statement

The original contributions presented in the study are included in the article/supplementary material. Further inquiries can be directed to the corresponding author.

## Ethics statement

Written informed consent was obtained from the individual(s) for the publication of any potentially identifiable images or data included in this article.

## Author contributions

SS: Conceptualization, Methodology, Project administration, Supervision, Visualization, Writing – original draft, Writing – review & editing. SD: Data curation, Formal analysis, Investigation, Writing – original draft. JL: Conceptualization, Formal analysis, Investigation, Supervision, Visualization, Writing – review & editing. SP: Data curation, Investigation, Supervision, Writing – review & editing. JR: Data curation, Formal analysis, Investigation, Project administration, Writing – review & editing. RG: Conceptualization, Investigation, Methodology, Project administration, Resources, Software, Supervision, Validation, Visualization, Writing – original draft, Writing – review & editing.

## References

[1] Chusid MJ Dale DC West BC Wolff SM. The hypereosinophilic syndrome: analysis of fourteen cases with review of the literature. Med (Baltimore). (1975) 54:1–27. doi: 10.1097/00005792-197501000-00001 1090795

[2] Ogbogu PU Rosing DR Horne MK 3rd. Cardiovascular manifestations of hypereosinophilic syndromes. Immunol Allergy Clin North Am. (2007) 27:457–75. doi: 10.1016/j.iac.2007.07.001 PMC2048688 17868859

[3] Parrillo JE Borer JS Henry WL Wolff SM Fauci AS. The cardiovascular manifestations of the hypereosinophilic syndrome. Prospective study of 26 patients, with review of the literature. Am J Med. (1979) 67:572–82. doi: 10.1016/0002-9343(79)90227-4 495628

[4] Sasano H Virmani R Patterson RH Robinowitz M Guccion JG. Eosinophilic products lead to myocardial damage. Hum Pathol. (1989) 20:850–7. doi: 10.1016/0046-8177(89)90096-8 2777241

[5] Weller PF Bubley GJ. The idiopathic hypereosinophilic syndrome. Blood. (1994) 83:2759–79. doi: 10.1182/blood.V83.10.2759.2759 8180373

[6] Mankad R Bonnichsen C Mankad S. Hypereosinophilic syndrome: cardiac diagnosis and management. Heart. (2016) 102:100–6. doi: 10.1136/heartjnl-2015-307959 26567231

[7] Ginsberg F Parrillo JE. Eosinophilic myocarditis. Heart Fail Clin. (2005) 1:419–29. doi: 10.1016/j.hfc.2005.06.013 17386864

[8] Fenoglio JJ Jr McAllister HA Jr Mullick FG. Drug related myocarditis. I. Hypersensitivity myocarditis. Hum Pathol. (1981) 12:900–7. doi: 10.1016/s0046-8177(81)80195-5 7298049

[9] Ogbogu PU Bochner BS Butterfield JH Gleich GJ Huss-Marp J Kahn JE. Hypereosinophilic syndrome: a multicenter, retrospective analysis of clinical characteristics and response to therapy. J Allergy Clin Immunol. (2009) 124:1319–25.e3. doi: 10.1016/j.jaci.2009.09.022 19910029 PMC2829669

[10] Groh M Masciocco G Kirchner E Kristen A Pellegrini C Varnous S. Heart transplantation in patients with eosinophilic granulomatosis with polyangiitis (Churg-Strauss syndrome). J Heart Lung Transplant. (2014) 33:842–50. doi: 10.1016/j.healun.2014.02.023 24709271

[11] Takkenberg JJM Czer LSC Fishbein MC Luthringer DJ Quartel AW Mirocha J. Eosinophilic myocarditis in patients awaiting heart transplantation. Crit Care Med vol. (2004) 32:714–21. doi: 10.1097/01.ccm.0000114818.58877.06 15090952

[12] Available at: https://www.uptodate.com/contents/treatment-and-prognosis-of-myocarditis-in-adults.

[13] Kuang F Li Amy DK. Biologic agents for the treatment of hypereosinophilic syndromes. J Allergy Clin Immunol In Pract vol. (2017) 5:1502–9. doi: 10.1016/j.jaip.2017.08.001 PMC5718167 29122152

[14] Zhong Z Yang Z Peng Y Wang L Yuan X. Diagnosis and treatment of eosinophilic myocarditis. J Transl Autoimmun. (2021) 4:100118. doi: 10.1016/j.jtauto.2021.100118 35005589 PMC8716607

[15] Brambatti M Matassini MV Adler ED Klingel K Camici PG Ammirati E. Eosinophilic myocarditis: characteristics, treatment, and outcomes. J Am Coll Cardiol. (2017) 70:2363–75. doi: 10.1016/j.jacc.2017.09.023 29096807

[16] Available at: https://www.fda.gov/news-events/press-announcements/fda-approves-first-drug-treat-group-rare-blood-disorders-nearly-14-years.

[17] Venkata Anuradha Samavedi MBBS. Hypereosinophilic Syndrome Treatment & Management: Approach considerations, surgical care, glucocorticoids. In: Hypereosinophilic Syndrome Treatment & Management: Approach Considerations, Surgical Care, Glucocorticoids. (2022). Available at: https://emedicine.medscape.com/article/202030-treatment.

[18] Korczyk D Taylor G McAlistair H May S Coverdale A Gibbs H. Heart transplantation in a patient with endomyocardial fibrosis due to hypereosinophilic syndrome. Transplant vol. (2007) 83:514–6. doi: 10.1097/01.tp.0000251385.71296.27 17318086

[19] Bondue A Carpentier C Roufosse F. Hypereosinophilic syndrome: considerations for the cardiologist. Heart (British Cardiac Society). (2022) 108:164–71. doi: 10.1136/heartjnl-2020-317202 34172539

